# Entering the Misinformation Age: Quality and Reliability of YouTube for Patient Information on Liposuction

**DOI:** 10.1177/22925503211064382

**Published:** 2021-12-06

**Authors:** Sahil Chawla, Jeffrey Ding, Leena Mazhar, Faisal Khosa

**Affiliations:** 1 8166 Faculty of Medicine, University of British Columbia, Vancouver, BC, Canada; 2 8167 Faculty of Science, University of British Columbia, Vancouver, BC, Canada; 3 8167 Department of Radiology, Vancouver General Hospital, Vancouver, BC, Canada

**Keywords:** liposuction, YouTube, patient education, educational quality, reliability, Éducation des patients, fiabilité, liposuccion, qualité de l’éducation, YouTube

## Abstract

**Background**: YouTube is currently the most popular online platform and is increasingly being utilized by patients as a resource on aesthetic surgery. Yet, its content is largely unregulated and this may result in dissemination of unreliable and inaccurate information. The objective of this study was to evaluate the quality and reliability of YouTube liposuction content available to potential patients. **Methods**: YouTube was screened using the keywords: “liposuction,” “lipoplasty,” and “body sculpting.” The top 50 results for each term were screened for relevance. Videos which met the inclusion criteria were scored using the Global Quality Score (GQS) for educational value and the Journal of the American Medical Association (JAMA) criteria for video reliability. Educational value, reliability, video views, likes, dislikes, duration and publishing date were compared between authorship groups, high/low reliability, and high/low educational value. **Results**: A total of 150 videos were screened, of which 89 videos met the inclusion criteria. Overall, the videos had low reliability (mean JAMA score = 2.78, SD = 1.15) and low educational value (mean GQS score = 3.55, SD = 1.31). Videos uploaded by physicians accounted for 83.1% percent of included videos and had a higher mean educational value and reliability score than those by patients. Video views, likes, dislikes, comments, popularity, and length were significantly greater in videos with high reliability. **Conclusions**: To ensure liposuction-seeking patients are appropriately educated and informed, surgeons and their patients may benefit from an analysis of educational quality and reliability of such online content. Surgeons may wish to discuss online sources of information with patients.

## Résumé

**Historique** : YouTube est actuellement la plateforme la plus populaire en ligne. Les patients l’utilisent de plus en plus comme ressource en chirurgie plastique. Pourtant, son contenu est en grande partie non réglementé, ce qui peut entraîner la diffusion d’information peu fiable et inexacte. La présente étude vise à évaluer la qualité et la fiabilité du contenu de YouTube sur la liposuccion à la disposition de patients éventuels. **Méthodologie** : Les chercheurs ont fait une recherche dans YouTube à l’aide des mots-clés *liposuction, lipoplasty* et *body sculpting*. Ils ont examiné les 50 premiers résultats de chaque terme pour en déterminer la pertinence. Ils ont classé les vidéos qui respectaient les critères d’inclusion d’après le score de qualité globale (SGQ) pour leur valeur éducative et les critères du *Journal of the American Medical Association* (JAMA) pour la fiabilité des vidéos. Ils ont comparé la valeur éducative, leur fiabilité, le nombre de visionnements, les « J’aime », les « Je n’aime pas », la durée et la date de publication entre groupes d’auteur, la forte et la faible fiabilité et la valeur éducative élevée ou basse. **Résultats** : Les chercheurs ont visionné 150 vidéos, dont 89 respectaient les critères d’inclusion. Dans l’ensemble, elles avaient une faible fiabilité (score JAMA moyen  =  2.78, ÉT  =  1.15) et une faible valeur éducative (SQG moyen  =  3.55, ÉT  =  1.31). Les vidéos téléversées par des médecins formaient 83,1% du contenu et présentaient une valeur éducative moyenne et un score de fiabilité plus élevés que celles des patients. Les visionnements, les « J’aime », les « Je n’aime pas », les commentaires, la popularité et la durée étaient beaucoup plus élevés lorsque les vidéos avaient une forte viabilité. **Conclusions** : Pour que les patients qui veulent subir une liposuccion reçoivent la bonne information, les chercheurs et leurs patients peuvent profiter d’une analyse de la qualité et de la fiabilité du contenu en ligne. Les chirurgiens pourraient souhaiter parler des sources d’information en ligne avec leurs patients.

## Introduction

Suction-assisted lipectomy, commonly known as liposuction, is the most common cosmetic plastic surgery procedure performed for both men and women.^1^ The procedure removes adipose tissue from subcutaneous spaces and remains the gold standard for body contouring.^2,3^ In 2020, there were 296 601 liposuction procedures performed by surgeons, which represented a 9.6% increase from the prior year.^1,4^ Although complications resulting from liposuction are relatively uncommon and low risk compared to other procedures,^5^ patient education is a vital component to higher satisfaction reports and better outcomes overall.^6–8^ Comprehensive patient education is important for cosmetic procedures as expected results satisfaction are dependent on preoperative discussions.^9^

With the advent of the World Wide Web, the Internet has become a popular source of health information^10^ as 80% of American adults reported looking for health-related information online.^11^ While this may be a convenient avenue for information, the Internet is not a well-regulated source and may widely disseminate low-quality and inaccurate information.^12–14^ YouTube is currently the most popular online platform in America, on which users can share non-peer-reviewed short videos.^15^ Approximately 80% of women and 82% of men in the United States use YouTube. From this, age groups 36 to 50 and 51 to 70 make up 91% and 83% of US YouTube users, respectively.^15^ Given that the top surgery among both age groups is liposuction,^1^ it imperative to assess the quality of information available on this platform.

Approximately 80% of women and 82% of men in the United States use YouTube.^15^ Also, it is interesting to note that while liposuction is the top surgery by age groups 36 to 50 and 51 to 70 for both men and women,^1^ and that 91% and 83% of these age groups, respectively are using YouTube, it is imperative to assess this platform's quality of information.^15^ Previous studies have confirmed that patients utilize YouTube as a resource on aesthetic surgery^16^; however, videos of this nature are often of low quality and educational value.^17^ Patients may seek out this information prior to a consultation without disclosure to the physician out of a fear of their reaction.^18^ Given that YouTube continues to be the world's second most visited website (after Google) and that cosmetic procedures have increased 22% since 2000, it is necessary that patients have access to reliable and educational information.^19,20^

To our knowledge, the quality and reliability of YouTube for patient information on liposuction have not been investigated. The objective of this study was to evaluate the educational quality and reliability of YouTube liposuction content that is available to potential patients.

## Methods

### Statement of Ethics

This study was exempt from institutional review board approval as the data was extracted in its entirety from publicly available resources. The dissemination of results will not identify any individual or generate new forms of identifiable information.

### Data Collection from YouTube and Exclusion Criteria

The following keywords, “liposuction,” “lipoplasty,” and “body sculpting” were inputted into YouTube.com on May 19, 2021. The first 50 videos per keyword were selected for evaluation, for a total of 150 videos. For each video, the following information was extracted: number of views, likes, dislikes, comments, length of the video in minutes, date published, name of publisher, and a subscriber count of the publisher. Videos were eliminated from the data pool if they were in a non-English language, a duplicate result of a previous video, unrelated to liposuction, or made for the purpose of medical education for healthcare providers.

### Authorship, Purpose, Educational Value, and Reliability Assessments

The remaining videos were assessed for authorship and purpose. Video authorship was categorized as either patients, healthcare providers, or both. The purpose was categorized as personal experience (patient speaking on their experience with liposuction) or patient education (speaking strictly on the medical aspect of liposuction). The reliability of the video was assessed using the Journal of the American Medical Association (JAMA) benchmark criteria, which considers the disclosure of the author's credentials, affiliations, sponsorships, copyright, and sources (Table 1).^21^ The educational value was determined using the Global Quality Score (GQS), which evaluates the flow of the video, the depth of the topic covered, and the amount of content that could potentially be of use to the viewer (Table 2).^22^ High video reliability was defined as a JAMA benchmark of 4, with low video reliability defined as anything less than 4. High educational value was defined as a GQS of 4 and greater, with “low” educational value defined as anything less than 4.

**Table 1.** JAMA Benchmark Criteria.^21^

Criteria	Description
Authorship	Author and contributor credentials and their affiliations should be provided.
Attribution	All copyright information should be clearly listed, and references and sources for content should be stated.
Currency	The initial date of posted content and dates of subsequent updates to content should be provided.
Disclosure	Conflicts of interest, funding, sponsorship, advertising, support, and video ownership should be fully disclosed.

Abbreviation: JAMA, Journal of the American Medical Association.

**Table 2.** Global Quality Scale (GQS) Criteria.^22^

Score	Description
1	Poor quality and unlikely to be useful for patient education
2	Poor quality and of limited use to patients because some information is present
3	Suboptimal quality and flow. May be useful to patients however, important topics are missing
4	Good quality and flow. Useful to patients because majority of important topics are included
5	Excellent quality and flow. Is highly useful to patients

### Data Analysis

Data analysis was performed using IBM SPSS Statistics (version 25). Continuous data were expressed by median, mean, and standard deviation. Categorical data were reported by absolute count and percentage. The significance cutoff was set as an alpha of 0.05. Mann-Whitney *U*-tests were run to determine differences in continuous dependent variables (views, age, popularity, length, likes, dislikes, and comments) between video reliability (low or high), educational value (low or high), and video type (patient education or personal experience).

## Results

**Figure 1.**
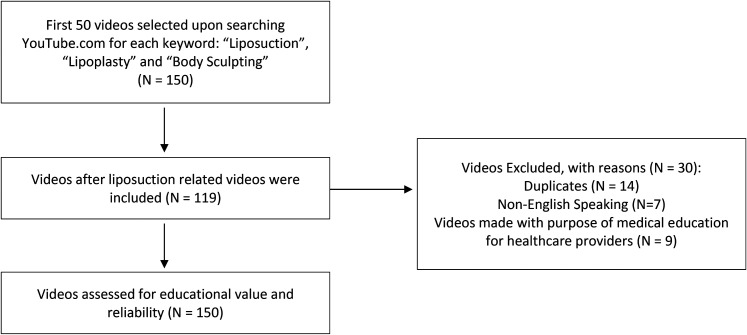
Flow Diagram for YouTube video selection. Performed in mid-May 2021.

Our analysis included 89 videos on liposuction, of which 80 (89.9%) were intended for patient education and 9 (10.1%) depicted personal experience (Figure 1). Healthcare providers were the predominant source of information (*N*  =  74; 83.1%), and videos uploaded solely by patients were uncommon (*N*  =  7; 7.9%). The videos collectively garnered 7 628 226 views and contained 6.3 h of content. On average, they had 85 710 views, 451 likes, 37 dislikes, 72 comments, 256 s of screen time, 1455 days on YouTube, and a popularity of 100 views/day. The mean JAMA and GQS scores were 2.78 (SD, 1.15) and 3.55 (SD, 1.31), respectively.

Table 3 presents a comparison of characteristics between videos of low versus high reliability/educational value. Views (*P*  =  .001), likes (*P* < .001), dislikes (*P*  =  .001), comments (*P* =  .001), popularity (*P*  =  .001), and length (*P*  =  .002) were significantly greater in videos with high reliability (JAMA benchmark criteria). No such differences at a statistically significant level were detected between videos of low versus high educational value (GQS criteria).

**Table 3.** Comparison of Video Characteristics by Reliability and Educational Value.

Video reliability (JAMA benchmark criteria)					
	Low (*N* *=* 57)		High (*N* = 32)		
	Median (Range)	Mean (SD)	Median (Range)	Mean (SD)	*P*
Views	7327 (3-466 268)	50 063 (100 819)	57 569 (82-1 021 881)	14 9207 (220 471)	.001
Age^a^	1015 (27-4565)	1506 (1297)	1205 (131-4226)	1364 (1033)	.962
Popularity^b^	8.9 (0-1022)	70 (162)	79.7 (0-864)	154 (210)	.001
Duration^c^	141 (6-1196)	207 (237)	233 (78-1581)	342 (334)	.002
Likes	41 (0-4300)	298 (695)	337.5 (0-3200)	714 (848)	<.001
Dislikes	3 (0-147)	20 (36)	19 (0-534)	68 (112)	.001
Comments	2 (0-499)	41 (100)	44 (0-923)	122 (195)	.001
Educational value (GQS criteria)					
	Low (*N* = 40)		High (*N* = 49)		
	Median (Range)	Mean (SD)	Median (Range)	Mean (SD)	*P*
Views	7004.5 (4-1 021 881)	69 811 (173 591)	29 727 (3-711 949)	98 690 (150 271)	.094
Age^a^	878.5 (27-4565)	1377 (1288)	1254 (72-4487)	1519 (1142)	.366
Popularity^b^	13 (0-864)	80 (159)	27.7 (0-1022)	117 (202)	.299
Duration^c^	142.5 (6-1196)	208 (232)	186 (15-1581)	295 (313)	.081
Likes	50 (0-1900)	270 (428)	199.5 (0-4300)	598 (952)	.074
Dislikes	3 (0-320)	27 (58)	13 (0-534)	46 (89)	.116
Comments	4 (0-923)	72 (179)	22.5 (0-542)	72 (123)	.219

Abbreviations: JAMA, Journal of the American Medical Association; GQS, Global Quality Score; SD, standard deviation.

*Notes*: “High” video reliability  =  JAMA benchmark of 4. “Low” video reliability  =  JAMA benchmark of 0 to 3. “High” educational value  =  GQS of 4 or 5. “Low” educational value  =  GQS of 1 to 3.

^a^ Days elapsed from time of upload to data collection.

^b^ Views divided by video age (n/day).

^c^ Denoted in seconds.

Table 4 contains a breakdown of characteristics for videos intended for patient education and personal experience. There were no statistically significant differences observed between the 2 video types.

**Table 4.** Comparison of Characteristics Between Patient Education and Personal Experience Videos.

	Patient education (*N* = 80)		Personal experience (*N* = 9)		
	Median (Range)	Mean (SD)	Median (Range)	Mean (SD)	*P*
Views	18243.5 (3-1 021 881)	80 126 (153 242)	67 276 (65-711 949)	135 354 (222 950)	.231
Age^a^	1205 (27-4565)	1533 (1240)	733 (72-1364)	765 (444)	.111
Popularity^b^	19.3 (0-864)	81 (148)	99.4 (0-1022)	271 (344)	.086
Duration^c^	180 (18-1265)	247 (245)	85 (6-1581)	329 (521)	.298
Likes	91.5 (0-3200)	389 (677)	852 (0-4300)	991 (1309)	.084
Dislikes	6.5 (0-320)	30 (55)	66 (0-534)	104 (168)	.080
Comments	13 (0-923)	61 (137)	59 (0-542)	162 (212)	.064

Abbreviation: SD, standard deviation.

^a^ Days elapsed from time of upload to data collection.

^b^ Views divided by video age (n/day).

^c^ Denoted in seconds.

## Discussion

Our analysis revealed that despite most liposuction video content being produced by health care providers, the mean JAMA score and GQS scores indicate low reliability and low educational quality, respectively. Various video characteristics, including video popularity and engagement, were significantly associated with video reliability (*P* = .01) but not educational content (*P* > .05). YouTube videos with higher educational quality and reliability are required to better educate patients on cosmetic liposuction procedures.

Patients are increasingly turning to YouTube to make more informed healthcare decisions. This is reflected in the 7.6 million views garnered by the 89 liposuction-related videos on YouTube which fit our inclusion criteria. The number of video views, popularity, length, likes, dislikes, and comments were significantly higher in videos with high reliability. This may be logically expected as highly reliable videos may be shared more within social circles and garner greater engagement from viewers. Surprisingly, no difference was observed in video views, popularity, length, likes, dislikes, and comments with educational value. This may be because these parameters don’t correlate with video quality.^23^ Personal experience videos did not have a higher number of views (*P* = .231), popularity (*P* = .086), likes (*P *= .084) and comments (*P* = .064), compared to patient education videos. An explanation for this may include the considerably fewer personal experience videos (*n* = 9) compared to patient education videos (*n* = 80). Prior studies on aesthetic surgery procedures also noted that most uploaded YouTube videos were developed by physicians for patient's education.

Similar studies have been conducted in dermatology, orthopedics, nephrology, cardiology to evaluate the quality of information on YouTube, respective to their specialty.^24–27^ One study looked at the online content quality of the top non-surgical procedure in the United States, neurotoxin injectables.^17^ The authors concluded that the reliability and educational value of information on YouTube was considerably low.^17^ Similarly, the results of our study indicate that the overall reliability and educational quality of YouTube videos related to liposuction procedures are low.

With the globalization of online medical information, there are emerging social support networks aimed at making online information more accessible. While YouTube was primarily used for entertainment purposes, recent evidence suggests it may be changing to disseminate health information.^28^ Recent evidence in healthcare videos also challenges the long-held belief preached by entertainment media experts, “content is king.”^16^ This phrase implies that high-quality, interesting and relevant content will result in a successful video. However, studies suggest that a high-quality video is likely to have no difference in engagement, compared with less optimal videos.^16^ While this uncertainty may be problematic for healthcare providers, it is important that we produce valuable and accurate content, in order to continue prioritizing our patients, inside and outside the clinic.

This study has several limitations. First, YouTube is a dynamic library of videos and search results may change over time. The search algorithm may show varied results based on variables such as geographic location. Second, our content search was limited to YouTube only and does not encompass videos that are available solely on popular websites aimed at liposuction patient education. Third, this study utilized highly regarded but unvalidated educational quality and reliability assessment tools, GQS and JAMA criteria. Lastly, since only English-speaking videos were included for analysis, this reduced the generalizability of our results.

This study examines YouTube videos pertaining to liposuction, but patients may be using other websites to obtain their information. Future studies may wish to identify and evaluate the quality of liposuction procedural information on other popular websites. Future studies may choose to assess video quality with additional validated tools like the Video Power Index (VPI) and DISCERN (Quality Criteria for Consumer Health Information). Also, given that health care providers were the predominant source of information and yet videos had low educational quality and reliability, future studies may want to develop a set of recommendations/guidelines to assist healthcare providers when creating and promoting comprehensive videos on YouTube.

## Conclusion

Patients searching YouTube for videos on liposuction treatment will be presented with a vast amount of content consisting of overall low reliability and low educational quality. As YouTube continues to grow and become a more popular tool for patients to gain medical information, there is an onus on surgeons to assess the quality and reliability of online content. Given that a large number of videos are uploaded by physicians and are of low quality, physicians should strive to ensure that their uploaded content is of high quality and educational value in order to better educate their prospective patients. This will help manage patients’ preconceptions, expectations, and surgery satisfaction. To ensure that liposuction-seeking patients are well-educated, plastic surgeons may wish to discuss reliable and educational online sources of information with them.
